# An Account of Diseases in an Eastern District of London, from the 20th of April, to the 20th of May, 1811

**Published:** 1811-06

**Authors:** 


### *An Account of Diseases in an Eastern District of London, from the 20th of April, to the 20th of May, 1811.*

| ACUTE DISEASES. | |
|---|---|
| Typhus mitior | 9 |
| Ephemera | 4 |
| Febris intermittens | 3 |
| Peripneumonia notha | 4 |
| Rheumatismus acutus | 2 |

| CHRONIC DISEASES. | |
|---|---|
| Tussis | 10 |
| Dyspnœa | 6 |
| Tussis cum Dyspnœa | 12 |
| Pleurodyne | 4 |
| Phthisis Pulmonalis | 4 |
| Apoplexia | 1 |
| Cephalalgia | 3 |
| Vertigo | 5 |
| Amenorrhæa | 6 |
| Menorrhagia | 2 |
| Dysuria | 4 |
| Enterodynia | 3 |
| Herpes | 2 |
| Hæmorrhois | 4 |
| Ascites | 3 |
| Anasarca | 4 |
| Rheumatismus Chronicus | 8 |

| PUERPERAL DISEASES. | |
|---|---|
| Menorhagia Lochialis | 4 |
| Ephemera | 3 |
| Dolor post partum | 4 |
| Constipatio | 5 |
| Dysuria | 6 |

| INFANTILE DISEASES. | |
|---|---|
| Febris mesenterica | 3 |
| Pertussis | 6 |
| Convulsio | 2 |
| Ophthalmia | 3 |

During the last few weeks numerous instances of fever have occurred, and these chiefly of the Typhoid kind. This disease was a few years ago so frequent in its recurrence, and so alarming in its consequences, particularly in hospitals and other public institutions, as to engage a very considerable share of medical attention. For some years past, however, it has very seldom made its appearance.

It is not improbable that this has been, in some measure, owing to the very large increase of new buildings, some of which occupy the space on which stood many of those closely confined dwellings that were occupied by the poor, and amongst whom the disease in question was most rapidly propagated. The disease has now returned, but has appeared in most cases in its milder form, though there have been instances of its propagation amongst those who have attended upon the sick.

This disorder has approached in its usual manner. It has been introduced by a degree of shivering and chilliness which has been succeeded by an increased action of the heart and arteries, considerable pain and unpleasant confusion in the head. This has sometimes abated in its violence, and gradually subsided, but at others has proceeded to a delirium, discovered rather by a low incoherent muttering, than by any expressions of violence.

The cases here referred to terminated favourably. When the cordial plan was adopted, in the early stage of the fever, symptoms were rather aggravated than relieved. A gentle diarrhæa, which is often the occasion of alarm, has frequently proved highly salutary.

BOTANICAL

